# Supplementary material for: Identification of Freezing-Responsive microRNAs and Their Targets in Chinese Jujube by Small RNA and Degradome Sequencing
Source: Int J Mol Sci. 2026 Sep 4;27(17):7912. doi: 10.3390/ijms27177912 (PMC13566890; doi:10.3390/ijms27177912)
Supplement: Supplementary file 1 [file ijms-27-07912-s001.zip › Supplementary Material S2/novel_miR_169.pdf]

Provisional ID : novel_miR_169
Score total : 432.7
Score for star read(s) : 3.9
Score for read counts : 425.8
Score for mfe : 1.4
Score for randfold : 1.6
Score for cons. seed :
Total read count : 847
Mature read count : 822
Loop read count : 0
Star read count : 25

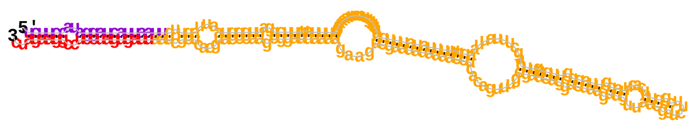

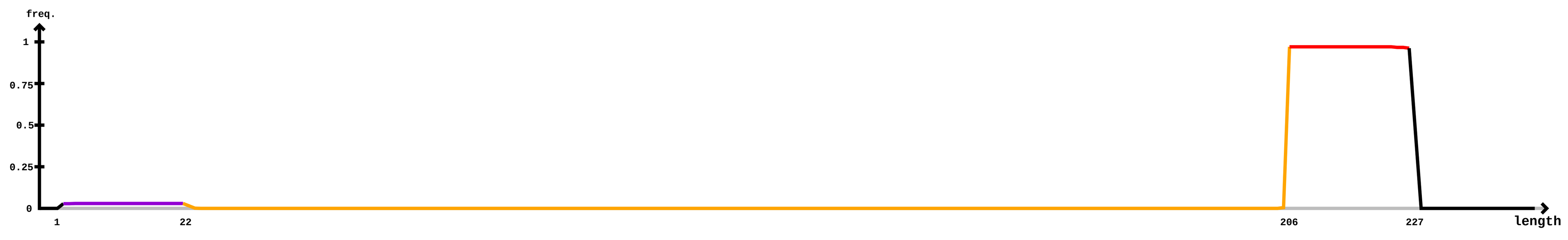

Star
Mature

```
5'- gaucuuccauaggaucauuaauuguugauuaaugggguuaggcuuguauuuuggaaaauuuaugggguuagguuuauauuuuuggaauuauuucgaguugaguugggauuggauucacuuuggcuucuggcaauuguugaaucuggacuagcguuugacacuccaaaauauaagcugaagaaaauuaggcgaacccaugcacucgcuaauuagugauccuccggaagaucauaauccaguuuggaaccaauu  -3'  obs
    gaucuuccauaggaucauuaauuguugauuaaugggguuaggcuuguauuuuggaaaauuuaugggguuagguuuauauuuuuggaauuauuucgaguugaguugggauuggauucacuuuggcuucuggcaauuguugaaucuggacuagcguuugacacuccaaaauauaagcugaagaaaauuaggcgaacccaugcacucgcuaauuagugauccuccggaagaucauaauccaguuuggaaccaauu        exp
    (((((((..((((((((((((((.(((...(((((((..(((((.(((((...............((((((((((((.(((.........(((.((((.(((((.(((.....(((.(....).)))..))).))))).)))))))........)))))))))))))))....))))))))))).)))))))....))).))))))))))))))..))))))))....((((...)))).......   reads  mm  sample
    ..ucuuccauaggaucauuaauu.....................................................................................................................................................................................................................................   2   0  LT-3
    ..ucuuccauaggaucauuaauu.....................................................................................................................................................................................................................................   10  0  JCK-3
    ..ucuuccauaggaucauuaauu.....................................................................................................................................................................................................................................   6   0  JCK-1
    ..ucuuccauaggaucauuaauu.....................................................................................................................................................................................................................................   1   0  JT-3
    ..ucuuccauaggaucauuaauu.....................................................................................................................................................................................................................................   1   0  JT-2
    ..ucuuccauaggaucauuaauu.....................................................................................................................................................................................................................................   4   0  JCK-2
    ....uuccauaggaucauuaauugu...................................................................................................................................................................................................................................   1   0  JCK-3
    ................................................................................................................................................................................................................auuagugauccuccggaag.........................   1   0  JCK-2
    ................................................................................................................................................................................................................Cuuagugauccuccggaagauc......................   1   1  JCK-3
    ................................................................................................................................................................................................................Cuuagugauccuccggaagauc......................   2   1  JCK-1
    .................................................................................................................................................................................................................uuagugauccuccggaag.........................   1   0  JCK-2
    .................................................................................................................................................................................................................uuagugauccuccggaag.........................   1   0  JCK-1
    .................................................................................................................................................................................................................uuagugauccuccggaagUu.......................   2   1  LT-2
    .................................................................................................................................................................................................................uuagugauccuccggaagau.......................   1   0  JCK-3
    .................................................................................................................................................................................................................uuagugauccuccggaagUu.......................   1   1  JCK-1
    .................................................................................................................................................................................................................uNagugauccuccggaagauc......................   1   1  JCK-2
    .................................................................................................................................................................................................................uuagugauccuccggaagauc......................   8   0  LT-1
    .................................................................................................................................................................................................................uuagugauccuccggaagauU.....................   2   1  JCK-3
    .................................................................................................................................................................................................................uuagugauccuccggaagauc......................   177 0  JCK-1
    .................................................................................................................................................................................................................uuagugauccuccggaagauc......................   220 0  JCK-3
    .................................................................................................................................................................................................................uuagugauccuccggaagauc......................   6   0  LT-3
    .................................................................................................................................................................................................................uuagugauccuccggaCgauc......................   1   1  JCK-2
    .................................................................................................................................................................................................................uuagugauccuccggaagCuc......................   1   1  JCK-1
    .................................................................................................................................................................................................................uuagugauUcuccggaagauc......................   1   1  JCK-2
    .................................................................................................................................................................................................................uuagugauccuccggaagauU.....................   1   1  JCK-2
    .................................................................................................................................................................................................................uuagugauccuccggaagauc......................   21  0  JT-2
    .................................................................................................................................................................................................................uuagugauccuccggaagauc......................   326 0  JCK-2
    .................................................................................................................................................................................................................uuCgugauccuccggaagauc......................   1   1  JCK-2
    .................................................................................................................................................................................................................uuagugaucAuccggaagauc......................   1   1  JCK-2
    .................................................................................................................................................................................................................uuagugauccuccggaagauc......................   23  0  JT-3
    .................................................................................................................................................................................................................uuagugauccuccggaagauc......................   8   0  LCK-3
    .................................................................................................................................................................................................................uuCgugauccuccggaagauc......................   1   1  JT-2
    .................................................................................................................................................................................................................uuagugauccuccggaagauU.....................   1   1  LT-3
```

```
 Star                                                                                                                                                                                                                Mature
gaucuuccauaggaucauuaauuguugauuaaaugggguuaggcuuguauuuuggaaaauuuaugggguuagguuuauauuuuuggaauuauuucgaguugaguugggauuggauucacuuuggcuucuggcaauuguugaaucuggacuagcguuugacacuccaaaauauaagcugaagaaaauuaggcgaacccaugcacucgcuaauuagugauccuccggaagaucauaauccaguuuggaaccaauu
..................................................................................................................................................................................................................uuagugauccuccggaagauc......................    12    0    LT-2
```
